# Production of IgGs with a human-like sialylation in CHO cells

**DOI:** 10.1186/1753-6561-9-S9-O3

**Published:** 2015-12-14

**Authors:** Céline Raymond, Anna Robotham, Maureen Spearman, Michael Butler, John Kelly, Yves Durocher

**Affiliations:** 1 Human Health Therapeutics Portfolio, National Research Council of Canada, Montreal, Quebec, H4P 2R2, Canada; 2 Department of Biochemistry and Molecular Medicine, University of Montreal, Montreal, Quebec, H3C 3J7, Canada; 3 Human Health Therapeutics Portfolio, National Research Council of Canada, Ottawa, Ontario, K1A 0R6, Canada; 4 Department of Microbiology, University of Manitoba, Winnipeg, Manitoba, R3T 2N2, Canada

## Background

IgGs that possess α2,6-sialylated Fc-glycans are thought to be responsible for the anti-inflammatory properties of intravenous immunoglobulins (IVIGs) [1], through a mechanism which has not been elucidated yet. The impact of this sialylation on the classic IgG's effector functions also remains unclear [2,3]. The understanding of these mechanisms has been impeded by the heterogeneity of the sialylated glycan species together with the relative rarity of α2,6-sialylated IgGs.

N-glycan sialylation is a post-translational modification where a sialic acid (SA) residue is added to the terminal galactose residue (Gal) of a growing glycan chain. The SA type (NANA or NGNA), the nature of the linkage between the SA and the Gal (α2,3 or α2,6), or the number of glycan antennae being sialylated, may vary according to the IgG subtype, the host cell in which it is expressed and the cell culture environment. A closer attention must thus be paid to the precise sialylation profiles leading to the reduced ADCC activity and to the gain of anti-inflammatory properties.

In humans, 10 to 15% of the circulating IgG1s are sialylated, carrying mostly complex di-antennary glycans with two Gal and one α2,6-linked SA residue (G2FS(6)1, where G stands for galactose, F for fucose and S(6) for α2,6SA). Most of the therapeutic monoclonal antibodies (mabs) are produced in Chinese hamster ovary (CHO) cells, which have a glycosylation machinery close to that of humans, but possess only α2,3-sialyltransferases (ST3) whereas humans have both α2,3- and α2,6-sialyltransferases. The Fc domain of mabs produced in CHO typically possesses N-glycans with low galactosylation and very low sialylation (0-2% of α2,3-sialylated glycans).

In this study, we show that the α2,6-sialylation of IgG1's Fc domain can be efficiently achieved by the transient coexpression of the human β1,4-galactosyltransferase 1 (GT) and α2,6-sialyltransferase 1 (ST6) in CHO cells, whereas the expression of one or the other glycosyltransferase alone does not significantly improve sialylation [4]. The process allows for the production of milligrams of human-like sialylated mabs within two weeks. We present a panel of four orthogonal assays for the fine characterization of the mabs' glycoprofile that are in very good agreement with each other.

## Experimental approach

CHO cells in suspension were transfected with polyplexes composed of polyethylenimine and plasmids encoding the mab and the enzymes. The proteins were expressed transiently, or stable pools were obtained following MSX selection in glutamine free medium. The mabs were purified on protein A resin from the supernatants after four days to avoid sialylation degradation (potentially caused by sialidases, pH and ammonia levels increase in late culture). The glycoprofiles were characterized through a set of four assays: lectin-blotting, capillary isoelectric focusing (cIEF), liquid chromatography coupled to electrospray ionisation mass spectrometry (LC-ESI-MS) and hydrophilic interaction liquid chromatography (HILIC). The relative glycan abundances obtained by LC-ESI-MS and HILIC correlated very well (Pearson coefficient 0.96). The evaluation of SA linkage type was accomplished by HILIC analyses after α2,3-specific or broad range sialidases treatment.

## Results and discussion

**Figure 1:**
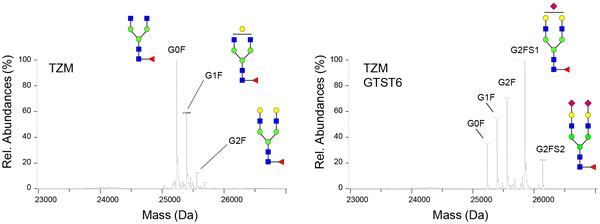
**LC-ESI-MS molecular weight profiles obtained for Fc/2 glycopeptides show that the co-expression of the Trastuzumab (TZM) with GT and ST6 yields glycoprofiles where G2FS1 is predominant**.

The transient expression of both GT and ST6 resulted in IgG1s where G2FS(6)1 was predominant (Figure 1), and 88% of the SA were of α2,6 type. 75% of the glycan branches were galactosylated and 25% sialylated, in comparison with 23% and 1% respectively in IgG1s expressed alone. In contrast, the co-expression of GT or ST6 led to less than 5% of sialylated glycan antennae. While the low galactosylation level in IgG1+ST6 can explain this result, it is surprising in the case of IgG1+GT where 70% of the branches were galactosylated.

Since heavily sialylated proteins were produced in CHO cells in our laboratory and others without cell engineering, we hypothesized that the glycan interactions with the Fc amino acids were limiting the access of ST3 to the galactosylated glycans. We thus expressed a mutated version of our IgG1, IgG1F243A, where the F243-glycan interaction is abolished, increasing the glycan exposition to the glycosyltransferases. Indeed, IgG1F243A was well galactosylated and sialylated: Gal and SA were present on 74% and 43% of the branches respectively. Upon GT expression, >95% of the antennae were galactosylated but the proportion of sialylated branches decreased to 33%. Therefore, the F243A mutation, while enhancing galactosylation and sialylation in general, did not promote endogenous sialylation of the Gal available in IgG1F243A+GT. The poor accessibility of the glycan to ST3 in the wild-type Fc was thus not sufficient to explain the lack of α2,3-sialylation in IgG1+GT, opening questions about the ST3 ability to use Gal provided by the human GT.

With this approach, the IgG1s were produced at yields around 15 mg/L. In order to reach yields closer to 200 mg/L, stable pools of CHO cells expressing GT, ST6 and an IgG1 were selected. However, the high mab productivity was achieved to the detriment of the sialylation level. Transfection and selection parameters were thus modified (new ST6 coding plasmid, increased proportion in the transfection mix) to reach high sialylation levels, along with mab concentrations around 100 mg/L.

Our method allows the fast production of milligrams of IgGs with a human-like Fc-sialylation, providing material for further functional studies, and also initiating the development of a recombinant substitute for anti-inflammatory IVIGs. The association of Fc/2 analysis by LC-ESI-MS with glycan analysis by HILIC constitutes a highly reliable platform for the fine characterization of antibodies' glycoprofiles.
